# Immunological Memory Transferred with CD4 T Cells Specific for Tuberculosis Antigens Ag85B-TB10.4: Persisting Antigen Enhances Protection

**DOI:** 10.1371/journal.pone.0008272

**Published:** 2009-12-14

**Authors:** Darragh Duffy, Amina Dawoodji, Else Marie Agger, Peter Andersen, Jürgen Westermann, Eric B. Bell

**Affiliations:** 1 Immunology Section, University of Manchester, Manchester, United Kingdom; 2 Department of Infectious Disease Immunology, Statens Serum Institut, Copenhagen, Denmark; 3 Institute for Anatomy, University of Lübeck, Lübeck, Germany; New York University, United States of America

## Abstract

**Background:**

High levels of death and morbidity worldwide caused by tuberculosis has stimulated efforts to develop a new vaccine to replace BCG. A number of *Mycobacterium tuberculosis* (*Mtb*)-specific antigens have been synthesised as recombinant subunit vaccines for clinical evaluation. Recently a fusion protein of TB antigen Ag85B combined with a second immunodominant TB antigen TB10.4 was emulsified with a novel non-phospholipid-based liposomal adjuvant to produce a new subunit vaccine, investigated here. Currently, there is no consensus as to whether or not long-term T cell memory depends on a source of persisting antigen. To explore this and questions regarding lifespan, phenotype and cytokine patterns of CD4 memory T cells, we developed an animal model in which vaccine-induced CD4 memory T cells could transfer immunity to irradiated recipients.

**Methodology/Principal Findings:**

The transfer of protective immunity using Ag85B-TB10.4-specific, CD45RB^low^ CD62L^low^ CD4 T cells was assessed in sub-lethally irradiated recipients following challenge with live BCG, used here as a surrogate for virulent *Mtb*. Donor T cells also carried an allotype marker allowing us to monitor numbers of antigen-specific, cytokine-producing CD4 T cells in recipients. The results showed that both Ag85B-TB10.4 and BCG vaccination induced immunity that could be transferred with a single injection of 3×10^6^ CD4 T cells. Ten times fewer numbers of CD4 T cells (0.3×10^6^) from donors immunised with Ag85B-TB10.4 vaccine alone, transferred equivalent protection. CD4 T cells from donors primed by BCG and boosted with the vaccine similarly transferred protective immunity. When BCG challenge was delayed for 1 or 2 months after transfer (a test of memory T cell survival) recipients remained protected. Importantly, recipients that contained persisting antigen, either live BCG or inert vaccine, showed significantly higher levels of protection (p<0.01). Overall the numbers of IFN-γ-producing CD4 T cells were poorly correlated with levels of protection.

**Conclusions/Significance:**

The Ag85B-TB10.4 vaccine, with or without BCG-priming, generated TB-specific CD4 T cells that transferred protective immunity in mice challenged with BCG. The level of protection was enhanced in recipients containing a residual source of specific antigen that could be either viable or inert.

## Introduction

Tuberculosis remains one of the world's leading causes of mortality and morbidity [1] despite the widespread use of the BCG vaccine (*Mycobacterium bovis*, bacille Calmette-Guérin). The BCG vaccine confers protection during childhood [2] and in some populations was found to have an efficacy of about 50% for up to 60 years[3]. Despite the beneficial effects, BCG vaccination does not prevent substantial problems with pulmonary disease in the adult population, especially in many developing countries [1]. There is an urgent need for a new vaccine. As a result of technological advances, increases in funding and heightened interest within the scientific community, there are a number of candidate vaccines in various stages of clinical trials [4]. The current candidate vaccines are broadly divided into live mycobacterium-based preparations, viral vectors or synthetic subunit vaccines. One strategy for developing new live vaccines is to introduce highly immunogenic, but non-pathogenic, epitopes from *Mycobacterium tuberculosis* (*Mtb*) into the BCG genome or alternatively to use a mutated *Mtb* in which at least two virulence genes have been altered so as to render the microorganism harmless [5]. Another option is to express *Mtb* antigens in the modified vaccinia virus Ankara (MVA) as a vaccine to boost subjects previously immunised with BCG [6]. The third approach aims to develop vaccines comprised of highly immunogenic *Mtb* antigens that can be combined with novel adjuvant systems. The present study is concerned with the latter type of vaccine.

A new vaccine strategy will only replace BCG when it is shown that it exceeds the degree of protection and safety currently provided by BCG. In the immediate future it is likely that any new vaccine, especially subunit/vector vaccines, will be used to augment the BCG-induced immune response. Attempts to boost BCG-generated immunity with a second dose of BCG have failed in both human trials and animal models [7], [8]. The reasons for this are not fully understood [1]. A heterologous prime/boost immunisation procedure – BCG followed by a subunit vaccine – is currently the favoured strategy to overcome the limitations of the BCG vaccine.

Recently, recombinant Ag85B was combined with a second *Mtb*-specific antigen TB10.4 [9], [10] that is strongly recognised by T cells from both BCG-vaccinated and TB patients [9]. A fusion protein consisting of Ag85B and TB10.4 was therefore produced and evaluated as a potential tuberculosis subunit vaccine [11]. It was shown that Ag85B-TB10.4, when combined with a cationic liposome adjuvant, induced a powerful cell-mediated response [12], IFN-γ producing CD4 T cells and protective immunity in mice challenged with *Mtb* [13].

It is often assumed that immunological memory depends on populations of memory CD4 T cells that are long-lived. This assumption was questioned by adoptive transfer experiments, reported several decades ago, that observed a rapid loss of memory in recipients that were not challenged immediately with specific antigen [14]. It has been argued that long-lived immunological memory depends on a source of persisting antigen [15], [16]. This issue remains unresolved and has important implications for vaccine development.

The present paper describes an experimental model that could assist in evaluating immunological memory induced by candidate vaccines. The study showed that protective immunity against a BCG challenge was adoptively transferred with a subset of CD4 T cells primed by BCG and boosted with the candidate vaccine Ag85B-TB10.4. In addition, the investigation showed that persisting recombinant vaccine could enhance the level of protection.

## Materials and Methods

### Ethics Statement

All animals were handled in strict accordance with good animal practice as defined by the UK Animal (Scientific Procedures) Act 1986. All animal work was approved by the University of Manchester Ethics Committee.

### Animals

B6 (C57BL6J, CD45.2) mice were purchased from Charles River, Manston, UK. A colony of the congenic strain B6.SJL (C57BL6Ly5.1, CD45.1) was established in SPF (specific pathogen-free) conditions from breeding pairs supplied by Charles River. Both strains were maintained under conventional husbandry in the Biological Services Unit of the University of Manchester. Mice used as recipients were given a sub-lethal dose (7Gy) of gamma irradiation delivered by an industrial X-ray tube, model MXR-320/26, at a dose rate of 0.76 Gy/min.

### Antigens and Adjuvants

The Danish 1331 strain of BCG was stored at −80°C and thawed before injection. The candidate subunit vaccine Ag85B-TB10.4, is a recombinant fusion protein composed of two antigens from *Mtb* (Ag85B-TB10.4) [11] that are expressed by BCG. The adjuvant CAF01 (dimethyl dioctadecyl ammonium bromide/trehalose 6,6′-dibehenate) [12], [17] was prepared by the lipid film method previously described [12] and stored at 4°C as a stable solution. The vaccine was prepared just before use. Each dose contained 2 µg of antigen mixed with 0.2 ml of adjuvant (250 µg DDA and 50 µg TDB).

### Immunisations

Mice were antigen-primed by injecting BCG or Ag85B-TB10.4 subcutaneously (s.c.) in both flanks, s.c. on the back of the neck and intraperitoneally (i.p.). A dose of 5×10^4^ CFUs BCG was divided between the different sites. For Ag85B-TB10.4 priming, mice received 3 injections 2 weeks apart. BCG immunised mice were boosted by injecting BCG or by distributing 2 µg Ag85B-TB10.4 combined with adjuvant between the same sites 2 mo after BCG priming.

### Adoptive Transfer

**Figure 1:**
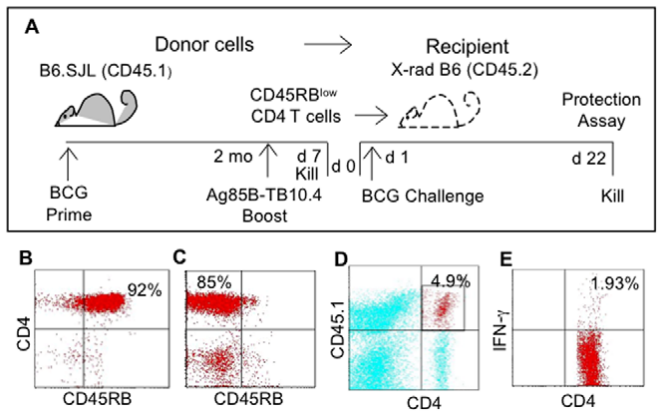
The development of an adoptive transfer model to evaluate *Mtb*-specific CD4 T cells. (A) Donor mice (B6.SJL, CD45.1) injected with BCG 2 mo before were boosted with Ag85B-TB10.4 and killed 7 days later. Spleen and lymph nodes were removed, depleted of B cells, CD8 T cells and macrophages. A representative profile of the resulting CD4 T cell population stained for CD45RB is shown (B). CD4 T cells were also depleted of CD45RB^high^ cells and injected into recipients on day 0 (d 0). A representative profile of CD45RB^low^ CD4 T cells used for adoptive transfer is shown (C). One day after transfer, irradiated recipients (X-rad) were challenged i.v. with live BCG, killed 3 weeks later (d 22) and spleens tested for a reduction in BCG numbers (Protection assay, CFUs/spleen). An aliquot of spleen cells was cultured overnight in the presence of Ag85B-TB10.4 antigen and stained for ICC IFN-γ. Donor-derived CD4 T cells (CD4^+^ CD45.1^+^) were gated in R1 (D) and analysed for IFN-γ positive cells (E). The donor-derived CD45.1^+^ CD4^−^ cells in profile D (upper left quadrant) were predominantly contaminating B cells. Numbers in the representative profiles are the percentage of events in the quadrant.

Immunised mice were killed, spleens and lymph nodes (mesenteric, inguinal, and auxiliary) removed, teased apart with forceps and filtered through a nylon monofilament mesh filter. Splenic red blood cells were removed by layering the cell suspension onto Histopaque (Sigma, Poole, UK) followed by centrifugation. Cells remaining at the interface were recovered, washed with PBS/FCS (phosphate buffered saline/2% foetal calf serum), pooled and viable cells counted using an electronic Scharfe CASY1 counter. Cells were stained for 30 min on ice with a cocktail of the following rat anti-mouse mAbs (clone in parentheses): CD45RB (16A), CD8 (53-6.7), CD19 (1D3), MHC II (M5/114.15.2), F4-80 all from BD Biosciences UK and CD34 (MEC14.7) from Serotec Ltd, Oxford, UK. A sample of stained cells was removed and incubated with FITC goat anti-rat IgG for analysis before depletion. Goat anti-rat IgG Biomag® beads (Metachem Diagnostics Ltd, Piddington, UK) were washed, mixed with the cells at a concentration of 2 mls of beads per 10^8^ cells and incubated with occasional re-suspension for 30 min on ice. The cell/bead suspension was placed on a strong magnet and the ferrous beads with cells attached were removed by magnetic adhesion. The cells that remained in suspension were recovered and washed twice with PBS/FCS. The cells were counted, a sample stained to assess the purity of the separation (as shown in Fig. 1C) and the concentration adjusted for injection. Control CD4 T cells were purified from naïve mice as described above but without the addition of mAb CD45RB. Cells were analysed by flow cytometry (FACSCalibur, BD, Cowley, UK) using Cell Quest-Pro software. All cell populations were injected i.v.

### BCG Protection Assay

Mice were injected i.v. in the lateral tail vein with 5×10^4^ BCG in 0.2 ml to assess the ability of recipients to eliminate the microorganism. Three weeks after injection spleens were removed, weighed and a fraction removed for cytokine and flow cytometric analysis. The remaining tissue was stored frozen to determine the number of surviving BCG at a later time. Frozen spleens were thawed, macerated on stainless-steel mesh, serially diluted, plated onto Middlebrook 7H11 agar and incubated for 18 to 21 days at 37°C. Colonies of BCG were counted and the results scored as CFU/whole spleen.

### Intracellular Cytokine (ICC) Analysis

Spleen cells (5×10^6^/ml) were cultured overnight in RPMI plus 2% FCS at 37°C with or without 5 µg/ml of Ag85B-TB10.4. Brefeldin A (2.25 µg/ml) was added for the last 4 hr of culture. Cells were washed, stained for CD4 (GK1.5) and donor CD45.1 (A20) or host CD45.2 (104) origin. The cells were washed, fixed with 4% paraformaldehyde and stained with anti-IFN-γ in 0.1% Saponin. Cells were analysed by flow cytometry.

### Statistical Analyses

Differences between means were assessed using Student's *t* test.

## Results

### Adoptive Transfer of Primed CD4 T Cells Protects against BCG Challenge

To examine the protective efficacy of vaccine-induced CD4 T cells, an adoptive transfer model was developed as illustrated in Fig. 1A. Donor B6.SJL mice were immunised with BCG and boosted 2 mo later with BCG or Ag85B-TB10.4. Previous experience suggested that antigen-primed CD4 T cells could be concentrated in the CD45RB^low^ subset [18]. Spleen and lymph node cells, enriched for CD4T cells (Fig. 1B), were depleted of the CD45RB^high^ population and the purified CD45RB^low^ CD4 T cells (Fig. 1C) transferred i.v. to sub-lethally irradiated recipients. The day after cell transfer, recipients were challenged i.v. with live BCG and the spleen assessed 21 days later for a reduction in BCG CFU. Since immunity to *Mtb* has been correlated with a Th1 IFN-γ response, spleen cell were stimulated *in vitro* with Ag85B-TB10.4 antigen and stained for intracellular cytokine (ICC) IFN-γ. Following transfer, allotype-marked CD45.1 donor T cells were identified in recipients by FACS (Fig. 1D), electronically gated and examined for ICC IFN-γ (Fig. 1E).

**Figure 2:**
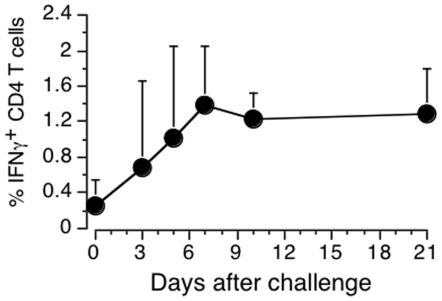
Kinetics of the antigen-specific CD4 T cell response in BCG-immunised mice after a boost with Ag85B-TB10.4. Mice were killed at selected days following antigen injection and spleen cells analysed for ICC IFN-γ. Results are the net percentage of IFN-γ^+^ CD4 T cells stimulated in the presence of Ag85B-TB10.4 after subtraction of the response without antigen. The results were pooled from 8 experiments. Each point represents the mean+SD of 3 or 4 mice, except day 3, n = 2.

The kinetics of the antigen-specific CD4 T cells was studied in BCG-primed mice that were boosted *in vivo* with Ag85G-TB10.4. Fig. 2 shows the percentage of ICC IFN-γ^+^ CD4 T cells at various times after boost. The response reached its maximum by day 7 and this time point was used for collecting CD4 T cells in subsequent experiments.

**Figure 3:**
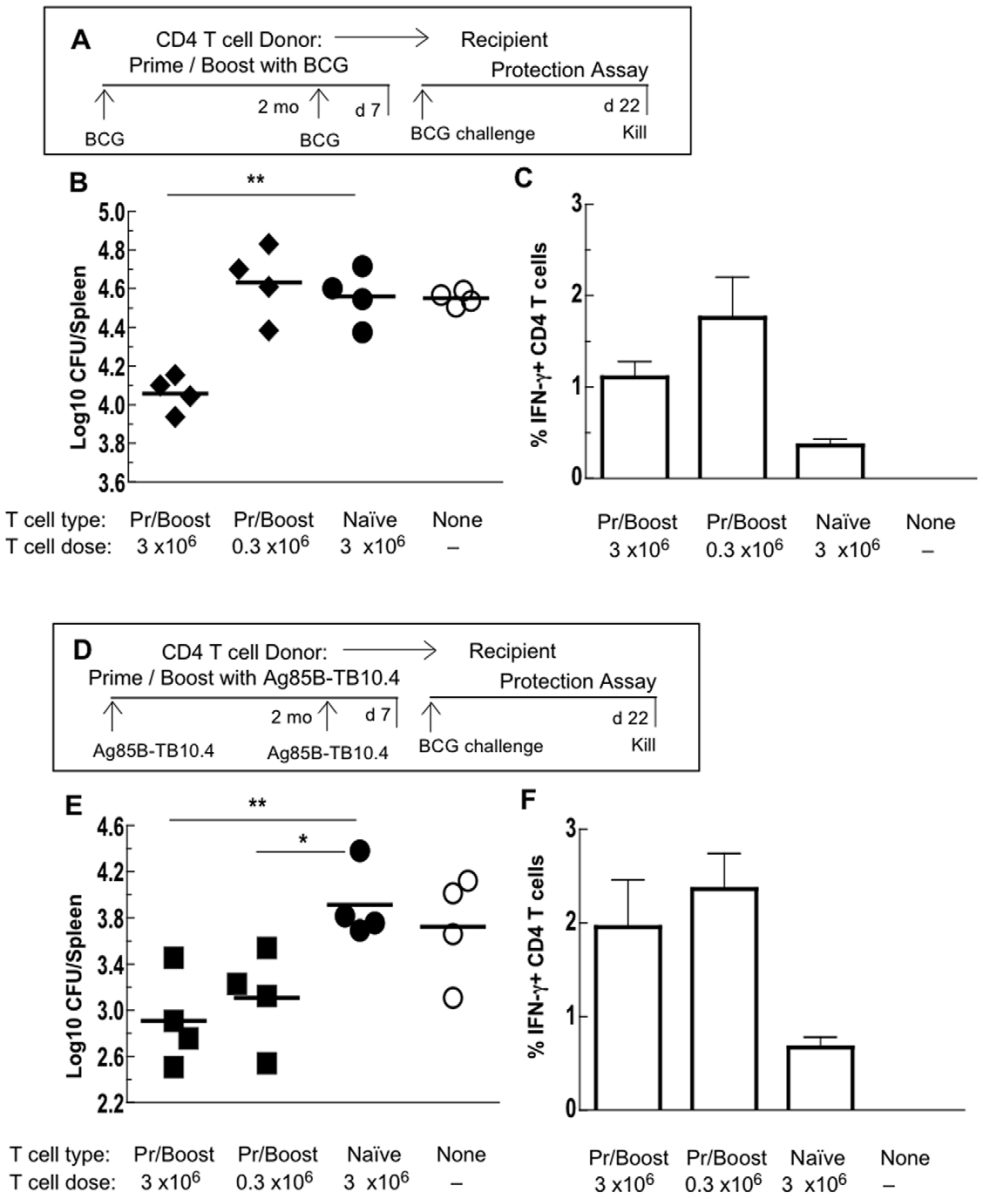
CD4 T cells specific for BCG or for Ag85B-TB10.4 transfer protective immunity. (A) CD45RB^low^ CD4 T cells were purified from donors primed and boosted with BCG (Pr/Boost) and injected (3×10^6^ or 0.3×10^6^) into irradiated recipients (B, C). Control recipients received 3×10^6^ unprimed (Naïve) CD4 T cells or no cells (None). (D) CD45RB^low^ CD4 T cells were purified from donors primed with Ag85B-TB10.4 and boosted with Ag85B-TB10.4 and 3×10^6^ or 0.3×10^6^ T cells injected into irradiated recipients. Control recipients received 3×10^6^ unprimed (Naïve) CD4 T cells or no cells (None). All recipients in experiments A and D were challenged i.v. with BCG the day after transfer, killed 3 weeks later and assayed for BCG CFU/spleen (B, E). Each point represents a single recipient. Donor-derived splenic CD4 T cells stimulated *in vitro* with Ag85B-TB10.4 were stained for intracellular cytokine (ICC) IFN-γ (C, F). The results were from single experiments. Horizontal bars or histograms + SD are means of 4 recipients/group. * p<0.05; ** p<0.01.

Initially we asked whether CD45RB^low^ CD4 T cells from donors that were primed with BCG and boosted with BCG could protect irradiated recipients against a live BCG challenge (Fig. 3A). Both high (3×10^6^) and low (0.3×10^6^) doses of CD45RB^low^ CD4 T cells were transferred. Significant protection (p<0.01) was transferred with the high dose of primed CD4 T cells but not the low dose (Fig. 3B). Nor was any protection transferred with a high dose of unprimed naive CD4 T cells. Donor T cells were also examined for the number of Ag85B-TB10.4-specific IFN-γ^+^ T cells by ICC staining (Fig. 3C). By necessity the cytokine levels were measured 3 weeks after bacterial challenge during the ongoing elimination of the BCG infection. Curiously, the number of antigen-specific IFN-γ^+^ CD4 T cells did not correlate with the level of protection. To assess the effectiveness of the recombinant vaccine in the absence of BCG, CD45RB^low^ CD4 T cells were purified from donors immunised with Ag85B-TB10.4 and boosted with Ag85B-TB10.4 (Fig. 3D). Significant protection was transferred with both 3×10^6^ – almost a log reduction (p<0.01) – and ten-fold fewer (0.3×10^6^) Ag85B-TB10.4-primed CD4 T cells (p<0.05) (Fig. 3E). Numbers of IFN-γ^+^ donor CD4 T cells were highest in those recipients that showed protective immunity, however, there were also significant numbers of cytokine-producing cells in mice receiving naïve T cells where there was no immunity (Fig. 3F).

### BCG/Ag85B-TB10.4 (Prime/Boost) CD4 T Cells Transfer Long-Lived Immunity

**Figure 4:**
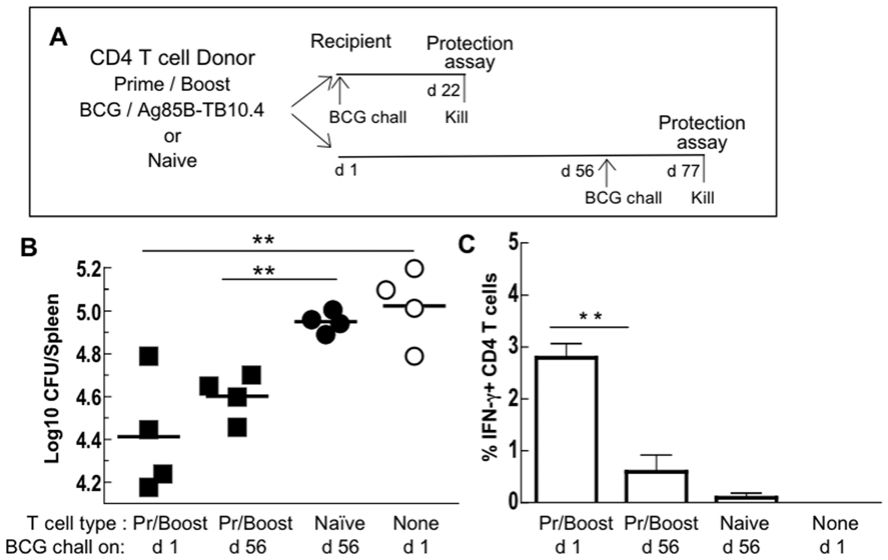
Protective immunity persists for at least 8 weeks following transfer of prime/boost CD4 T cells. (A) CD45RB^low^ CD4 T cells (3×10^6^) from donors primed with BCG and boosted with Ag85B-TB10.4 (Pr/Boost) or 3×10^6^ CD4 T cells from unprimed donors (Naïve) or no cells (None) were transferred to irradiated recipients and challenged with BCG immediately (d 1) or 8 weeks later (d 56) (B, C). Three weeks after challenge spleen cells were assayed for BCG CFUs (B) and ICC IFN-γ^+^ donor CD4 T cells (C). The results are from one experiment. Each point represents a single recipient. Horizontal bars or histograms + SD are means of 4 recipients/group. ** p<0.01.

Since BCG vaccination has been used worldwide, any new vaccine would need to provide protection in individuals previously immunised with BCG. Therefore, CD4 T cells were assessed in BCG-primed mice boosted with Ag85B-TB10.4. CD45RB^low^ CD4 T cells were purified from BCG-primed/Ag85B-TB10.4-boosted donors and transferred to irradiated recipients. In addition, we wanted to assess whether the antigen-specific T cells survived for long periods after transfer as a measure of immunological memory. Hence, recipient mice were challenged immediately after transfer (d 1) or after a delay of 2 mo (Fig. 4A, d 56). The results showed that CD4 T cells from BCG primed/Ag85B-TB10.4 boosted donors (Pr/Boost) transferred significant protection (p<0.01) to the BCG challenge administered immediately after transfer (Fig. 4B). To determine whether immunological memory persisted, CD45RB^low^ CD4 T cells from BCG/Ag85B-TB10.4 (Pr/Boost) donors were injected into irradiated recipients and the BCG challenge delayed for 8 weeks (Fig. 4A). Recipients in which the BCG challenge was delayed (Fig. 4B, d 56) remained protected (p<0.01) confirming the continued survival of CD4 T cells responsible for immunity. The level of protection was similar to that in recipients challenged immediately after transfer (p<0.01); there was no significant difference (p>0.2) between groups challenged on d 1 or d 56 (Fig. 4B). The experiment was repeated with the same result (data not shown). Despite the long-lived protection, there were significantly more (p<0.01) IFN-γ producing CD4 T cells detected in the spleen of recipients challenged immediately following T cell transfer than in recipients in which the challenge was delayed (Fig. 4C).

It is known that a lymphopenic environment such as that induced following irradiation will enhance the survival and expansion of the donor T cell population [19], [20]. Therefore, as an additional test of transferred immunity, CD45RB^low^ CD4 T cells were injected into normal non-irradiated instead of irradiated hosts. CD4 T cells from BCG/Ag85B-TB10.4 (Pr/Boost) donors successfully transferred immunity to non-irradiated recipients although, as expected, the level of protection was lower (log CFU/spleen: Pr/Boost, 3.59±0.02; No cells, 3.88±0.11; p<0.05).

### Persisting Antigen Enhances Protection

**Figure 5:**
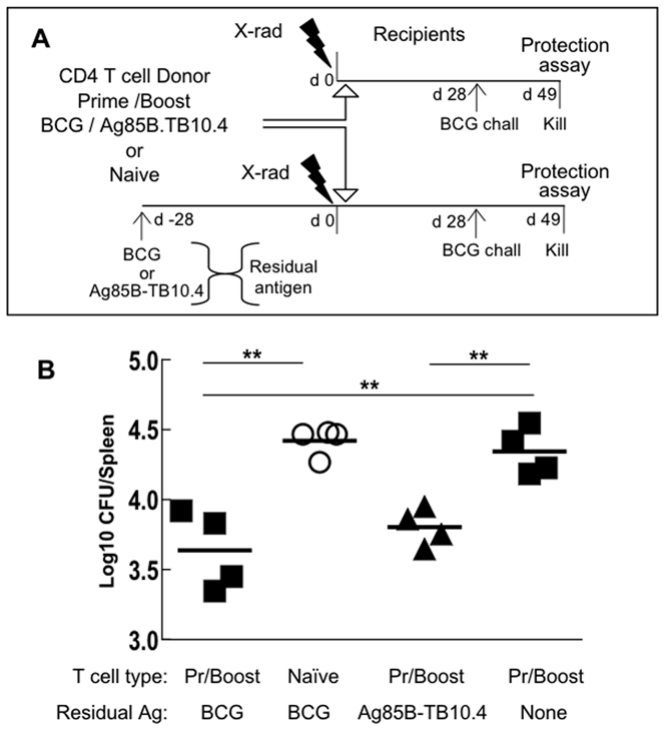
Persisting antigen enhances protection. (A) To establish a potential source of residual antigen (Ag) mice were injected with BCG or Ag85B-TB10.4, 4 weeks (d -28) before irradiation and cell transfer. Control recipients received no source of residual Ag (None) and were irradiated 1 day before cell transfer. CD45RB^low^ CD4 T cells (3×10^6^) from BCG primed/Ag85B-TB10.4 boosted donors (Pr/Boost, solid symbols) or unprimed CD4 T cells (Naïve, open symbols) were transferred into recipients after irradiation on d 0. All recipients were challenged with BCG 4 weeks later (d 28). (B) Three weeks after challenge spleen cells were assayed for BCG CFUs. Each point represents a single recipient. Horizontal bars are means of 4 recipients/group. ** p<0.01.

The role of residual antigen in maintaining immunological memory remains controversial. We explored the influence of antigen in the adoptive transfer model and asked whether protective immunity was prolonged or shortened by the presence of persisting antigen. To generate a source of residual antigen, recipient mice were pre-injected (Fig. 5A, d -28) with either BCG or Ag85B-TB10.4 and irradiated 4 weeks later, immediately prior to cell transfer. Irradiation destroys the host's own immune response leaving behind a potential source of residual antigen. The object was to compare Ag85B-TB10.4, a non-viable antigen, with live BCG as a source of persisting antigen. As in the previous experiment recipient mice received BCG/Ag85B-TB10.4 (Pr/Boost) CD45RB^low^ CD4 T cells (Fig. 5B, solid symbols) and the BCG challenge was delayed, in this case, for one month (Fig. 5A, d 28). A control group containing residual BCG (i.e. injected with BCG and irradiated 4 weeks later) received naïve instead of primed CD4 T cells (Fig. 5B, open symbols). The protection in recipients with persisting antigen, both BCG and Ag85B-TB10.4, was significantly increased (p<0.01) compared with that seen in antigen-free recipients or in recipients that received naïve CD4 T cells (Fig. 5B). A second experiment in which BCG was the source of persisting antigen gave a similar result (data not shown). These observations suggested that persisting antigen augmented the protective immunity transferred by *Mtb*-specific CD4 T cells.

## Discussion

The development of a new vaccine to supplement or replace BCG is an international priority. To make the investigation of vaccine-induced immunological memory to TB more accessible, we wanted to establish an animal model suitable for laboratories without class III (P3) containment facilities. Hence, we opted to challenge recipients with live BCG rather than *Mtb*. Aside from the clear difference in virulence, *Mtb* and BCG are very similar – both are intracellular pathogens controlled predominantly by CD4 T cells. In the more advanced stages of infection, *Mtb* appears to induce a subset of CD8 T cells not seen after BCG vaccination [21], but the role of these CD8 T cells in protection has never been formally demonstrated. In the first stages of infection (the focus of the present study), it is clear that both BCG and *Mtb* are controlled by CD4 T cells. Furthermore, the new recombinant vaccine Ag85B-TB10.4 used in the present investigation expresses two immunodominant antigens common to both BCG and *Mtb*. This vaccine has been shown to induce protective immunity in mice infected with *Mtb* [11]. Hence, BCG should serve at this stage as a reliable surrogate for *Mtb* challenge.

Previous studies established that antigen-specific CD4 T cells become concentrated in a small subset of CD4 T cells identified by a surface phenotype linked with antigen-priming, namely CD45RB^low^ CD44^high^ CD62L^low^ [22], [23]. This subset represents <10% of the CD4 T cell population and may be routinely purified for adoptive transfer [18], [24]. An allotype marker was used to allow identification of donor T cells in recipients several months after transfer to irradiated recipients. Following sub-lethal irradiation the lymphocyte population of the host becomes temporarily destroyed, creating a lymphopenic environment and initiating homeostatic proliferation [19], [25] that encourages donor cell engraftment.

Protective immunity to BCG challenge was successfully transferred with CD45RB^low^ CD4 T cells from BCG/BCG and BCG/Ag85B-TB10.4, prime/boost donors. The number of CFU/spleen was consistently reduced by approximately a half-log. The same number of CD4 T cells (3×10^6^) from donors that were immunised exclusively with Ag85B-TB10.4 (primed and boosted with Ag85B-TB10.4), transferred a greater level of protective immunity (∼1-log reduction in CFU). Furthermore, significant protection was also transferred by one-tenth this number (0.3×10^6^) of the CD4 T cells.

The apparent difference in protection using T cells from mice immunised with BCG or solely with Ag85B-TB10.4 was unexpected and suggests there could be quantitative differences in the number of antigen-specific T cells transferred and/or qualitative differences. A recent study from our laboratory analysing CD44^high^ CD4 T cells from C57BL/6 mice immunized with BCG was compared with those from mice immunised with a subunit vaccine (Ag85B-ESAT-6) [13]. The latter is very similar to the one used here; both recombinant vaccines were combined with the same CAF01 adjuvant. In mice immunised with Ag85B-ESAT-6 compared with BCG there were significant differences in the type of multifunctional T cells (i.e. those producing more than one of the cytokines IFN-γ/IL-2/TNFα) present in mice immunised more than 10 months earlier. CD4 T cells from these mice (analogous to Ag85B-TB10.4/Ag85B-TB10.4 prime/boost T cells used here) were predominantly of the triple- (∼60%) and double-producing IL-2^+^/TNFα^+^ type (∼25%) with negligible levels of single IFN-γ-producing T cells. In contrast, T cells from mice immunised with BCG and stimulated with the BCG extract PPD (purified protein derivative) (T cells similar to that from BCG/BCG prime/boost donors used here) had far fewer triple-producing T cells (∼35%), a different type of double-producing (∼35% INF-γ^+^/TNFα^+^) and likewise few single IFN-γ-producing T cells (∼3%). Triple-producing multifunctional T cells have been shown to correlate better with protective immunity than single-producing T cells in several disease models [26]–[28]. It remains to be confirmed whether such a difference in quality of CD4 memory T cells could explain the differences in levels of protection observed in mice immunised with BCG or solely with Ag85B-TB10.4.

Modest but significant levels of immunity (p<0.05) were also transferred to normal non-irradiated recipients. The lower level of immunity was expected since the donor T cells would compete less well for survival against the dominant *in situ* host population [29], [30]. Antigen-primed CD4 T cells not only provided immediate protection, but also conferred immunity in recipients challenged months after cell transfer – a measure of immunological memory. Finally, the investigation showed that persisting antigen, viable and inert, enhanced the protective immunity transferred by *Mtb*-specific CD4 T cells.

The CD45RB^low^ CD62L^low^ CD4 T cells used here to transfer immunity are frequently described as both “memory” cells responsible for long-lasting immunity and confusingly, “effector” cells – cytokine-producing terminally differentiated lymphocytes [31]. Paradoxically, the CD45RB^low^ subset is known to be short-lived [22], [32]–[34]. It is also known that CD45RB^low^ CD4 T cells may return to a resting “naïve” phenotype (CD45RB^high^ CD62L^high^) in the absence of stimulation [33]–[36], a resting T cell with a long lifespan [22], [32]–[34]. It was critical, therefore, to show that the *Mtb*-specific CD4 T cells survived and were not simply short-lived effector cells. Hence, *Mtb*-specific T cells were “parked” in recipients in the absence of antigen for 2 mo before BCG challenge. The mice remained protected showing that the *Mtb-*specific population survived. Based on other studies many of the transferred T cells would have re-expressed the CD45RB^high^ molecule associated with a long-lived quiescent state [18], [37]–[39]. It was argued elsewhere [15] that immunological memory accrues from the initial clonal expansion, the legacy of which is a high frequency of long-lived antigen-specific T cells. This concept was strongly supported by experiments showing that protective immunity to *Mtb* was adoptively transferred with CD45RB^high^ CD44^low^ CD62L^high^ CD4 T cells (i.e. a naïve phenotype) from donors immunised to *Mtb* from which the mycobacterium had been destroyed months before [37].

In the present experiments, the number of IFN-γ^+^ Ag85B-TB10.4-specific CD4 T cells was poorly correlated with protection; the levels were at best a crude predictor of transferred immunity. Other groups also reported that ELISPOT and ICC values for IFN-γ had a poor correlation with protection [40]–[43]. The reasons for this are not entirely clear. One possibility is that the traditional ICC and ELISPOT assays may not be identifying all antigen-specific T cells responsible for memory. The studies by Mohrs *et al* [44] indicate that in order for CD4 T cells to synthesise and release cytokines (effector T cells), “2-hits” with specific antigen are required – the first to generate cytokine message, the second to initiate synthesis of the cytokine protein. These 2 antigen encounters are likely to be separated in time and in tissue location. Hence, naïve T cells primed in lymph nodes will only synthesise cytokines when they re-encounter antigen, for example, at the site of infection. The current assays, which stimulate T cells *in vitro* (2nd hit), will only induce cytokine production in those T cells that have already been primed. Importantly, many of the CD4 T cells initially primed following immunisation may fail to find antigen a second time and may therefore return to a resting state. A change from CD45RB^low^ to CD45RB^high^ following transfer was confirmed in the present study (unpublished observations; DD, EBB). Such cells become phenotypically and functionally indistinguishable from naïve T cells [35], [39]. This memory population would not be detected by current *in vitro* assays. Furthermore, as mentioned earlier, multifunctional CD4 T cells appear to be more reliable indicators of protective immunity than assays that screen for IFN-γ alone [26]–[28].

The role of persisting antigen in maintaining CD4 T cell memory remains a contentious issue [16], [45], [46]. Evidence supporting a vital role for residual antigen continues to accumulate [47]–[50]. The numbers of successful live vaccines that provide long-lasting protection (e.g. smallpox, measles, mumps, rubella, polio and others) is also consistent with a role for persisting antigen. The present studies explored two different types of residual antigen – a live source that could replicate (BCG) or an inert protein that could only decline with time (Ag85B-TB10.4). Both types of residual antigen were equally effective in enhancing protective immunity. We can only surmise that relevant epitopes from both the viable and non-viable sources were available to *Mtb*-specific CD4 T cells *in vivo*. In addition, given that the size of the memory response appears to be determined by the number of antigen-specific T cells [24], the amount of antigen presented was probably not the limiting factor. The enhancing effect of *live* BCG was consistent with earlier work. Fifty years ago it was reported that live but not killed BCG induced lasting protective immunity [51]. More recently, Olsen et al. [52] found that immunity measured in the spleen declined in BCG-immunised mice when the microorganism was cleared by antibiotic therapy. The augmented protection associated with residual recombinant vaccine was more surprising and implies a significant retention of specific antigenic epitopes, in all probability related to the delivery vehicle. Whether or not a non-viable vaccine will maintain memory may, therefore, depend on the nature of the adjuvant. Recent evidence indicates that the cationic liposome adjuvant DDA/TDB, used here, persists for an extended period at the injection site (D. Christensen, personal communication) and maintains immunological memory very effectively [13]. The success of non-viable vaccines may ultimately hinge on advances made in developing new delivery systems that are safe for use in humans and that release antigen slowly from a depot.
